# Study protocol: multimodal physiotherapy as an add-on treatment to botulinum neurotoxin type A therapy for patients with cervical dystonia: DysPT-multi—a prospective, multicentre, single-blind, randomized, controlled study

**DOI:** 10.1186/s13063-021-05705-8

**Published:** 2021-10-25

**Authors:** Christian Werner, Dana Loudovici-Krug, Steffen Derlien, Florian Rakers, Ulrich C. Smolenski, Thomas Lehmann, Norman Best, Albrecht Günther

**Affiliations:** 1 Department of Neurology, St. Georg Klinikum Eisenach, Eisenach, Germany; 2 Institute for Physiotherapy, Jena University Hospital, Jena, Germany; 3 Hans-Berger-Department of Neurology, Jena University Hospital, Jena, Germany; 4 Institute of Medical Statistics, Jena University Hospital, Jena, Germany

**Keywords:** Cervical dystonia, Spasmodic torticollis, Physical therapy, Physiotherapy, Botulinum neurotoxin, Treatment, Rehabilitation

## Abstract

**Background:**

Botulinum neurotoxin (BoNT) is currently the best therapeutic option in the treatment for cervical dystonia (CD). Additional treatments like physiotherapy (PT) may even improve the results of the BoNT injection with type A (BoNT-A), but there are no definite recommendations. In the last few years, some studies showed tendencies for PT as an adjuvant therapy to benefit. However, high-quality studies are required.

**Methods:**

This study is a multicentre, randomized, single-blind, controlled trial to demonstrate the effectiveness of a multimodal PT program compared to a nonspecific cupping therapy, additionally to the BoNT-A therapy. Two hundred participants will be assigned into the multimodal PT plus BoNT intervention arm or the BoNT plus cupping arm using randomization. Primary endpoint is the total Score of Toronto Western Spasmodic Rating Scale (TWSTRS). Secondary endpoints are the mobility of the cervical spine (range of motion, ROM), the TWSTRS subscales, and the quality of life (measured by questionnaires: CDQ-24 and SF-36). Patients will be single-blind assessed every 3 months according to their BoNT injection treatment over a period of 9 months.

**Discussion:**

The study aims to determine the effectiveness and therefore potential benefit of an additional multimodal physiotherapy for standardized treatment with BoNT-A in patients with CD, towards the BoNT-therapy alone. This largest randomized controlled trial in this field to date is intended to generate missing evidence for therapy guidelines.

**Trial registration:**

The study was registered in the German Clinical Study Register before the start of the patient recruitment (DRKS00020411; date: 21.01.2020).

## Administrative information

**Table:** 

Title {1}	Study protocol: Multimodal physiotherapy as an add-on treatment to botulinum neurotoxin type A therapy for patients with cervical dystonia DysPT-Multi: A prospective, multicentre, single-blind, randomized, controlled study
Trial registration {2a and 2b}.	German Clinical Study Register (DRKS00020411).
Protocol version {3}	1
Funding {4}	Ipsen Pharma
Author details {5a}	Christian Werner - St Georg Klinikum Eisenach, Department of Neurology Dana Loudovici-Krug – Jena University Hospital, Institute for Physiotherapy Steffen Derlien – Jena University Hospital, Institute for Physiotherapy Florian Rakers – Jena University Hospital, Department of Neurology Ulrich C. Smolenski – Jena University Hospital, Institute for Physiotherapy Thomas Lehmann – Jena University Hospital, Institute of Medical Statistics Norman Best – Jena University Hospital, Institute for Physiotherapy Albrecht Günther – Jena University Hospital, Hans-Berger-Department of Neurology
Name and contact information for the trial sponsor {5b}	Dr. med. A. Günther albrecht.guenther@med.uni-jena.de
Role of sponsor {5c}	role in study design, recruitment, interpretation, writing/ publication

## Introduction

### Background and rationale {6a}

Cervical dystonia (CD) usually occurs as primary adult-onset dystonia between 30 and 50 years. CD represents the largest part of primary dystonia syndromes with an estimated prevalence between 2.8 and 18.3 per 100,000 [1]. Dystonia is characterized by involuntary patterned contractions, which often lead to abnormal postures or movements [2]. In the case of CD, this affects cervical and neck muscles, which results in an abnormal head or neck position or motion [3]. The majority of all patients also suffer from pain [4] and further from non-motor symptoms like depression or social phobia [5, 6]. Therapeutic options are purely symptomatic and not causal, meaning that only an alleviating of the symptoms is possible. Therapy of choice is a selective peripheral denervation of the affected muscles using botulinum neurotoxin (BoNT). The BoNT “Abobotulinumtoxin A” is injected into the affected muscles in individual doses. This leads to a temporary weakening or paralysis of the corresponding muscles. The injection must therefore be repeated regularly at 3-month intervals. The BoNT therapy is effective, well-tolerated and safe for patient with CD [7, 8]. At present there are no specific recommendations for physiotherapy (PT) in CD [9, 10]. The effects of additional physiotherapy in CD-patients have only rarely been examined to date and the quality of available evidence is ranged from very low to low [11]. De Pauw et al. published a review of six clinical trials and ten case series [12]. However, the different physiotherapy techniques, duration of interventions, and outcome measures made it difficult to compare study data and results of these trials. Four of the studies were evaluated with a moderate or high quality by De Pauw et al. [13–16]. These four trials, on 20 to 40 patients, showed a trend towards a benefit of the physiotherapy program in the Toronto Western Spasmodic Rating Scale (TWSTRS) subscores, the SF-36 score, and the rotation deviation. De Pauw et al. concluded that multimodal physiotherapy concepts with the main focus on active exercises in combination with BoNT type A (BoNT-A) treatment probably have a beneficial effect on pain, severity of the CD, and activities of daily life. However, the authors report that further studies are required before any specific recommendations can be given [11, 12]. Under the assumption that add-on PT results in a better outcome, two RCTs examined a special PT technique (Bleton regime) vs. regular PT [17, 18]. The results showed that there were no significant differences between the different PT interventions, though both groups in each of these two studies showed improvements, especially in TWSTRS and generic quality of life after different add-on PT intervention [17, 18]. A recent pilot study with 18 patients was also able to show clear advantages of additional PT [19]. There were significant improvements in mental health (SF-36), in the rotation and flexion/extension dimension of the cervical spine ROM and in every TWSTRS subscore following PT intervention consisting of two PT sessions per week over a period of 12 weeks [19]. From the data available at present, it can be concluded that multimodal add-on PT, which focuses on active exercises, has an advantage in terms of pain, severity of CD, and activities of daily life compared to the sole BoNT therapy. However, in order to give clear recommendations, further, in particular RCTs with a large number of participants are required [9, 10, 18].

### Objectives {7}

The main objective is to determine the effect of the multimodal physiotherapy as an add-on treatment to BoNT-A therapy for patients with cervical dystonia on the disease expression of cervical dystonia. Secondary objectives are effects on the statics and mobility of the cervical spine and on disease-specific and disease-unspecific health perception.

### Trial design {8}

This study is planned as a multicentre, randomized, single-blind, controlled trial.

## Methods: participants, interventions and outcomes

### Study setting {9}

University Hospital, Outpatient clinic of the Department of Neurology / Institute for Physiotherapy, Jena, Germany

University Hospital, Centre for Neurodegeneration/ Dystonia outpatient clinic, Tübingen, Germany

Two more study centers are planned. All parties involved obtain the same study procedures and devices including the according training of the measurement.

### Eligibility criteria {10}

- Study population:
  - Adult patients diagnosed with CD
  - Already treated with BoNT therapy
- Inclusion criteria:
  - CD diagnosed, based on Col/Cap-classification [3]
  - Continuously treated with BoNT-A in approximately 3 months’ interval (11 to 13 weeks; aiming to prevent bias by using different BoNT-A-products with different dosing strategies)—independent of a special dilution or concentration of the BoNT
  - At least 12 months’ treatment without BoNT-A scheme changes over the last 3 injections
  - Signed patient informed consent
- Exclusion criteria:
  - CD with antecollis/-caput only
  - CD with dystonic tremor only
  - PT intervention within the last 3 months before study onset (previous PT is possible, but not necessary for participation)
  - Contraindications for physiotherapy treatment
  - Contraindications for BoNT-A treatment

### Who will take informed consent? {26a}

The medical study director (principal investigator) of the respective study center will take informed consent.

### Additional consent provisions for collection and use of participant data and biological specimens {26b}

Not applicable.

### Interventions

#### Explanation for the choice of comparators {6b}

From the data available at present, it can be concluded that multimodal add-on PT, which focuses on active exercises, has an advantage in terms of pain, severity of CD and activities of daily life compared to the sole BoNT therapy. Participants will be assigned into the multimodal PT plus BoNT intervention arm or the BoNT plus cupping arm using randomization. Cupping therapy was chosen as comparator (control group) to compare with a technique, which includes the presence of a therapist using a passive application on not directly by cervical dystonia affected muscles. In addition, cupping therapy reduces chronic neck pain, which is common in CD patients [20]. However, there is no evidence that cupping therapy affects cervical spine mobility, disease severity, or quality of life in ZD patients. Owing to the nature of the PT intervention, therapists and participants cannot be blinded to treatment allocation. However, the rater assessing the TWSTRS will be blinded for the treatment group of the patient.

#### Intervention description {11a}

Study duration for every participant amounts 9 months which is divided into three periods of 3 months each. In the first period, both groups will obtain their usual BoNT treatment. In the second phase, the respective intervention starts. In this time, the intervention arm conducts the add-on multimodal PT program that includes 24 PT sessions while the control arm receives 24 sessions of an add-on unspecific cupping treatment. The third period contains 3 months BoNT treatment for both arms as follow-up period. The participants will be assessed every 3 months according to their BoNT injection intervals. That means there are four assessment time points: at the beginning of period one, at the start of the intervention period, at the end of the intervention period respectively the start of the third period, and at the end of the third period.

Patients can choose their own physiotherapists. Moreover, every physiotherapist receives a therapy recommendation (Table 1). They are not obliged to use every mentioned technique, but they should assign their therapy to different proposals with regard to the evaluation.

**Table 1:** Therapy recommendation of physiotherapy (intervention group)

Muscular	Neuromuscular	Peripheral approaches
□ Mobilization of the cervical spine (passive-active)	□ Multidimensional movement therapy: o with visual control o without visual control	□ Posture training
□ Isometric technique (of antagonists)		□ Perception training
□ Muscle relaxation (stretching etc., gentle)	□ PNF: diagonals of shoulder blades, upper extremity, head, etc.	□ Relaxation techniques
□ Treatment of trigger points (gentle)		□ Whole-body tension/ stem management
	□ Balance training	
	□ Coordination training	

##### Group A: Intervention: physiotherapy—24 therapy units, two times weekly for a duration of 30 minGroup B: Control: cupping therapy (dry, CT)

Target of the CT is hyperemia and metabolic activation for self-regulation of the connective tissue. Moreover, pain relief and relaxation are further goals of the CT. The maximum duration is about 15 min or in case of clear formation of a hematoma or by dropping of the glasses. The therapy shall be applied to the patients’ shoulder girdle, in particular the rotator cuff (*Musculus infraspinatus and supraspinatus*), the rhomboids (*Musculus rhomboideus major et minor*) and the chest muscles (*Musculus pectoralis major*).

#### Criteria for discontinuing or modifying allocated interventions {11b}

The DysPT-Multi study is an interventional study but not a clinical drug trial in humans. There are few risks associated with study therapy, but patients receive BoNT-A (not an investigational drug, but an interventional medicinal product) as standard. This concomitant medication carries known risks. As the study is conducted in accordance with the Declaration of Helsinki and following the ICH-GCP guideline, the establishment of a safety management with regard to the study therapy and the BoNT-A administration has been implemented. For this purpose, adverse events observed in the course of the clinical study will be recorded and evaluated by the study centers and regularly re-evaluated by the study director with regard to the causal relationship to the study therapy or the BoNT-A administration. In the context of individualized physiotherapeutic treatment, a high level of patient safety can be assumed. Adverse events are more likely to occur for standard therapy with BoNT-A.

#### Strategies to improve adherence to interventions {11c}

Physical therapy practices will be provided with contact information for study centers for consultation and follow-up.

#### Relevant concomitant care permitted or prohibited during the trial {11d}

Concomitant therapy or medication will be queried and noted. With regard to the intention-to-treat-analysis, the change of medication during the study duration will be noted as test plan deviation/ query. Nevertheless, the patient will stick to its study group until the study ends.

#### Provisions for post-trial care {30}

Not applicable.

### Outcomes {12}

Primary endpoint:

- Total score of the Toronto Western Spasmodic Rating Scale (TWSTRS)

Secondary endpoints:

- TWSTRS severity, disability, and pain score
- Mobility of the cervical spine using an inertial measurement unit (ROM)
- SF-36 to evaluate the generic health
- CDQ-24 to evaluate disease-specific quality of life [16]
- Subgroup analysis on age, severity, disease duration, BoNT-A treatment duration

### Participant timeline {13}

**Fig. 1:**
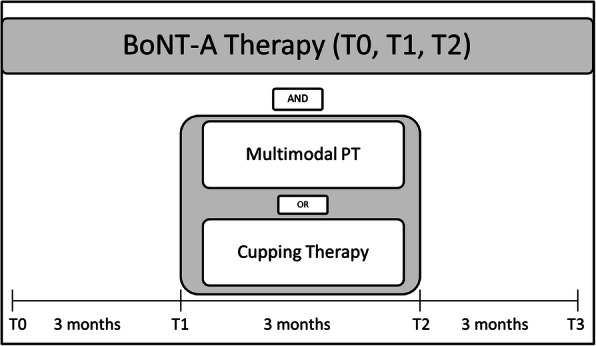
study timeline

Participant timeline is shown in Table 2 and Fig. 1

**Table 2:**
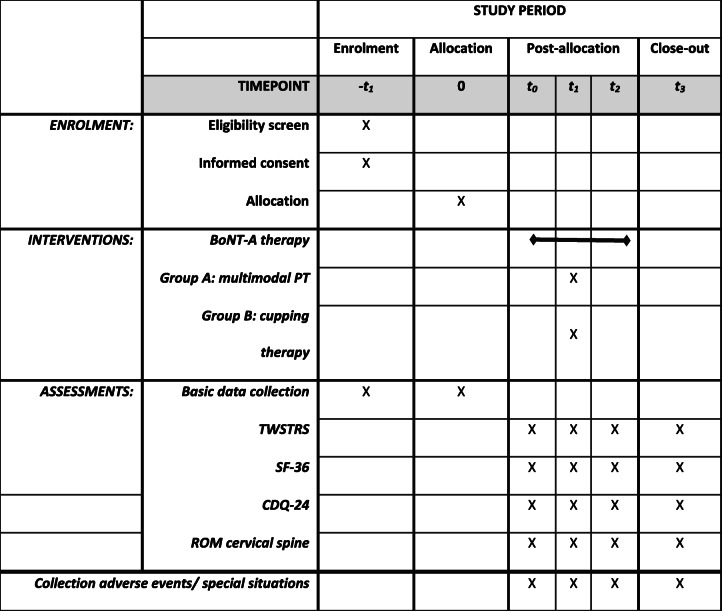
Schedule of enrolment, interventions, and assessments (SPIRIT figure)

.

### Sample size {14}

In the pilot trial [19], total TWSTRS reduced on average by 8.7 points after the PT plus BoNT intervention. Standard deviations for BoNT and PT plus BoNT intervention were 7.4 and 5.4 points, respectively. To detect a difference of at least three points between the two groups with a power of 90%, 100 patients per group should be analyzed. Assuming a drop-out rate of 10%, a total of 224 patients (112 per group) should be randomized in the trial.

### Recruitment {15}

The medical principal investigator of each study center recruits the patients in the outpatient clinic of the department of neurology.

### Assignment of interventions: allocation

#### Sequence generation {16a}

Computer-generated random number, generated by the software “Random Tool”

An Internet-based randomization tool provided by the Centre for Clinical Studies, University Hospital Jena, will perform the randomization. This will occur after the patient has signed the patient consent form, has met all inclusion and no exclusion criteria, and therefore has been included in the study.

#### Concealment mechanism {16b}

The group allocation is assigned by the medical study director of the respective study center. The patient obtains the respective treatment information to hand it over to their therapist.

#### Implementation {16c}

By the medical study director of the respective study center.

### Assignment of interventions: blinding

#### Who will be blinded {17a}

- Outcome assessors (for every patient is measured in the same way and there are no special assessments concerning the intervention group)

#### Procedure for unblinding if needed {17b}

Not applicable.

### Data collection and management

#### Plans for assessment and collection of outcomes {18a}

The assessors are especially trained before start of the recruitment and/ or were already experienced in the different measuring methods.

The primary endpoint of the study is the total score of the Toronto Western Spasmodic Torticollis Rating Scale (TWSTRS). The TWSTRS disease-specific scoring system was developed to describe the severity of CD [21]. This measurement tool is widely used and is explicitly recommended for comparing studies or performing meta-analyses [2, 12]. Validity and reliability of the score could be confirmed [21]. In this study, the TWSTRS is collected in a blinded manner by examination. The total score range is 0–85, with a high score representing greater expression or impairment [22].

Secondary endpoints are the TWSTRS subscales (severity, limitation of daily life, and pain), the range of motion (ROM) of the cervical spine assessed by three-dimensional motion analysis using inertial sensors (Company: Velamed), the SF-36 questionnaire [23] to assess generic health perception, and the CDQ-24 questionnaire [24] to assess disease-specific quality of life. Furthermore, a subgroup analysis by age, severity, duration of disease, and duration of BoNT treatment is planned.

The effect of an add-on multimodal PT will be investigated with regard to the individual subscales of the TWSRTS in order to present more specific effects, if necessary.

The clinical correlate of cervical dystonia is the cervical spine misalignment or malposition, including the adjacent muscles and joints. Thus, the statics and mobility of the cervical spine represent an important parameter for assessing the effect of the intervention. In order to be able to obtain data that are as objective as possible, the three-dimensional instrument-based measurement system (company: Velamed) determines the extent of cervical motion for this endpoint. This system is based on ultrasound measurement signals and can accurately determine movements in all three planes of motion. The validity and the reproducibility have been successfully tested [25]. Another measurement instrument with a similar technical basis and accurate motion analysis could already be evaluated in patients with CD and was recommended for diagnosis as well as for progress documentation [26]. Furthermore, a measurement system (company: Zebris) that works similarly to the three-dimensional motion measurement was successfully used for motion analysis in the pilot study [19]. However, the measurement with inertial sensors is more precise.

The SF-36 provides the study with a cross-disease score system to assess quality of life. Validity and reproducibility have been tested [23].

To assess disease-specific health perception, the CDQ-24 questionnaire is used. This questionnaire was developed to assess the quality of life of patients with CD and/or blepharospasm. The original questionnaire is available in German. Validity and reliability are available [24].

#### Plans to promote participant retention and complete follow-up {18b}

The patients are scheduled regularly by the corresponding outpatient clinic of the department of neurology and hence stick to the study protocol time line.

#### Data management {19}

The data collection serves scientific purposes. The data are generated at the participating study centers. All data required for the clinical study are entered by the corresponding staff in the study centers in a computer-based online data entry system and immediately transferred to the servers at the Centre for clinical Studies (CCS) Jena.

Data entry is performed via web application on the servers of the CCS of the University Hospital Jena using the study management software “OpenClinica®”. The software fulfills the regulatory requirements (GCP, 21CFRPart11). The data are collected via an encrypted data connection (HTTPS) in input masks via web browser. To ensure pseudonymized data analysis, each patient is assigned a unique patient identification number.

The study management software “OpenClinica®” is also used for data management. The plausibility of the data is checked by range, validity, and consistency checks. Non-plausible or missing data are queried at the study center. Every change to the data, e.g., due to the incorporation of answered queries, is documented in the database via an automatic change tracking (audit trail). The use of a hierarchical, role-based access concept makes unauthorized access to the data impossible.

As a documentation center, the CCS at Jena University Hospital is also responsible for data storage. The backup of electronic data occurs on a regular basis. The data storage facilities are located in a locked, central room to which only system administrators have access.

#### Confidentiality {27}

The anonymity of the data within the scope of evaluations is ensured. The assignment between patient and patient ID is made in the respective study center by the patient identification list and is not stored in the database. A daily complete backup of all data takes place. The patient identification list is stored in the corresponding study center folder (Investigator Site File, ISF).

#### Plans for collection, laboratory evaluation, and storage of biological specimens for genetic or molecular analysis in this trial/future use {33}

Not applicable.

### Statistical methods

#### Statistical methods for primary and secondary outcomes {20a}

The primary endpoint (TWSTRS total score) is evaluated using a linear mixed model. The intervention (specific physiotherapy or control therapy) is considered as a fixed factor and the study center as a random factor in the model. Furthermore, adjustment is made for baseline TWSTRS total score by including this variable as a covariate in the model. The significance level for the confirmatory test for treatment differences is 5%.

A linear mixed model will be fitted for each secondary endpoint. Intervention is modeled as a fixed factor and center as a random factor and the respective baseline variable is included as a covariate. All secondary end points will be compared exploratively at a 5% significance level.

(Intention-to-treat-analysis)

For all endpoints, group-specific descriptive statistics (means ± standard deviation) will be provided.

#### Interim analyses {21b}

These are not planned.

#### Methods for additional analyses (e.g., subgroup analyses) {20b}

All endpoints are additionally defined into subgroups and analyzed by age, severity of CD, and previously received PT.

#### Methods in analysis to handle protocol non-adherence and any statistical methods to handle missing data {20c}

The primary analysis will be intention-to treat, with all randomized patients included, regardless of adherence to the protocol. In case of significant protocol non-adherence, secondary analysis will be conducted to estimate per-protocol intervention effects.

#### Plans to give access to the full protocol, participant level-data and statistical code {31c}

Not applicable.

### Oversight and monitoring

#### Composition of the coordinating center and trial steering committee {5d}

It must be ensured that each person responsible for the documentation in the Case Report Form (CRF) can be identified. A list with signature and abbreviation of the persons who are allowed to make entries in the CRF (signature/delegation log) is filed in the study folder.

This overview also identifies other persons involved in the clinical trial with their names, signatures, and abbreviations as well as their responsibilities and authorities. A list with the signature and abbreviation of the persons is filed in the study folder.

#### Composition of the data monitoring committee, its role, and reporting structure {21a}

In accordance with guideline for Good Clinical Practice (ICH GCP E6), monitoring is established on a smaller scale for quality assurance purposes. The purpose of the monitoring of the clinical study is to ensure that the clinical study is conducted in accordance with the approved protocol and -the ethical regulations and standards are met. The study director commissions the CCS Jena with this task. Monitoring includes a central initiation/webinar to train and brief the study team prior to the start of recruitment and regular on-site visits as well as a final visit to properly close the study site. Monitoring will be performed according to the standard operating procedures of the CCS Jena. Detailed information on the scope, procedure, and contents of the monitoring as well as procedures to ensure data quality and necessary measures in case of protocol violations are described in a monitor manual to be released by the study director. The study physician will allow the monitor direct access to the original data and documents for study-related monitoring. The monitor is required to keep all information confidential and to uphold the study participants’ fundamental right to integrity and privacy.

#### Adverse event reporting and harms {22}

All adverse events (AEs) and reports of special situations will be recorded in the patient record during the course of the clinical trial and documented in the CRF in a timely manner. The Ethics Committee receives serious AEs once a year as a list after evaluation by the study director.

#### Frequency and plans for auditing trial conduct {23}

An audit serves as a systematic and independent review of the activities and documents related to the clinical study to determine whether the study-related activities are being conducted in accordance with the protocol, the standard operating procedures, and the applicable legal/ethical requirements. The study director may have an independent audit conducted at the participating institutions at any time as part of the quality assurance process. In this case, the study personnel or the participating institution will allow the auditor to inspect all documents necessary for the audit.

#### Plans for communicating important protocol amendments to relevantparties (e.g., trial participants, ethical committees) {25}

After a positive opinion has been issued prior to the start of the clinical trial, the responsible ethics committee must be notified, for example, of changes in the content of the study protocol (amendment) as well as the patient information and consent form, additional study centers, and changes in the study physician.

#### Dissemination plans {31a}

Efforts are underway to publish the results of this clinical trial in a prestigious international medical journal.

## Discussion

This study is generated to determine the effectiveness of an additional multimodal physiotherapy for standardized treatment with abobotulinumtoxin A in patients with CD. Presently, it is the largest single-blinded assessment study on this disease. The question to be answered is whether an additional multimodal PT program can improve the therapy of CD. As already described above, add-on PT has great potential for optimizing therapy for CD patients. To date, however, this has not been adequately investigated and should be examined in a large cohort for a clear recommendation. The current largest studies in this area examined specialized physiotherapy on the assumption that an add-on PT achieves a better outcome, although the actual effectiveness of an additional PT has not yet been investigated [17, 18]. Nevertheless, the two studies showed that both PT interventions had a presumably positive effect on severity and quality of life caused by CD.

As primary endpoint the TWSTRS will be used which is internationally well established. The questionnaires SF-36 and CDQ-24 are used extensively in similar studies [13–16]. These parameters can be used to determine the effectiveness of an add-on multimodal PT in various aspects of the disease and will make it easier to compare results with other trials. Moreover, an exact and objective measurement method, using an inertial measurement unit, is chosen for the ROM determination, instead of a simple angle measurement by an examiner. This method offers a number of advantages in contrast to conventional goniometry. The focus is on the exact measurement of degrees. Furthermore, the measuring system can also rule out evasive movements, record secondary movements while executing a movement, and document the movement sequence, so that, e.g., tremor movements can be objectified. Due to the good experience in the pilot study with a similar system, there is great potential for objective data and for other studies, planning research on CD severity [19].

The study is designed in such a way that the first phase of the study duration can determine disease manifestation under the usual BoNT therapy. The intervention takes place after 3 months, demonstrating the effect of the multimodal PT program over nonspecific cupping therapy as control group. After the endpoint at 6 months, a follow-up measurement continues after further three months to show possible long-term effects.

Physiotherapeutic sessions in an interval of two units of 30 min per week correspond to the current reality of the health system in Germany. Also with regard to the physiotherapeutic techniques, care was taken to create a therapy concept that physiotherapists can implement with the usual repertoire of treatment techniques. If a clearly better outcome can be achieved with an additional PT, the further implementation of the multimodal PT-program, as described in the study design, can be directly implemented [27].

## Trial status

Protocol version: 01

Recruitment start: 22.06.2020

Approximate date when recruitment will be completed: 2022

## Data Availability

The principal investigator, co-investigator, and statistician for the protocol will have access to and be responsible for all of the data for this study.

## Abbreviations

BoNT: Botulinum neurotoxin
BoNT-A: Botulinum neurotoxin type A
CD: Cervical dystonia
CDQ-24: Craniocervical dystonia questionnaire – 24
PT: Physiotherapy
ROM: Range of motion
SF-36: Short Form (36) Health Survey
TWSTRS: Toronto Western Spasmodic Rating Scale
